# From Epigenetic Regulation to Protein Degradation: Emerging Strategies for Anti-Infective Drug Discovery

**DOI:** 10.3390/ijms27093977

**Published:** 2026-04-29

**Authors:** Andressa Francielli Bonjorno, Diogo Boreski, Ana Luísa Rodriguez Gini, Pamela Souza Tada da Cunha, Jhonnathan Alves Moura, Chung Man Chin, Cauê Benito Scarim, Jean Leandro Dos Santos

**Affiliations:** 1 São Paulo State University (UNESP), School of Pharmaceutical Sciences, Araraquara 14800-903, Brazil; diogo.boreski@unesp.br (D.B.); ana.gini@unesp.br (A.L.R.G.); pamela.tada@unesp.br (P.S.T.d.C.);; 2 São Paulo State University (UNESP), Institute of Chemistry, Araraquara 14800-060, Brazil; jhonnathan.alves@unesp.br; 3 Advanced Research Center in Medicine (CEPAM), School of Medicine, Union of the Colleges of the Great Lakes (UNILAGO), São José do Rio Preto 15030-070, Brazil

**Keywords:** host–pathogen, PROTAC, BacPROTAC, LYTAC, AUTAC, ATTEC

## Abstract

Infectious diseases remain a major global health challenge, driven by antimicrobial resistance, pathogen persistence, and the limited integration of mechanistically innovative therapeutic approaches. Emerging evidence indicates that epigenetic regulation is fundamental to host–pathogen interactions, influencing transcriptional programmes associated with virulence, immune evasion, stress adaptation, and phenotypic plasticity. In organisms such as bacteria, parasites, and intracellular pathogens, including *Mycobacterium tuberculosis* and *Plasmodium falciparum*, chromatin-associated regulators and DNA-modifying enzymes have been identified as dosage-sensitive determinants of infection outcomes. Traditional strategies focus primarily on occupancy-driven enzymatic inhibition. In contrast, targeted protein degradation (TPD) introduces an event-driven pharmacological paradigm in which transient ligand engagement triggers sustained depletion of regulatory proteins. Platforms such as proteolysis-targeting chimeras (PROTACs) and BacPROTACs exemplify the ability to exploit host and pathogen proteolytic systems, thereby expanding the druggable proteome beyond conventional small-molecule targets. This review examines the relationship between epigenetic regulation and pathogen survival, highlights recent advances in degradation technologies, and discusses conceptual and translational challenges in implementing TPD in antimicrobial and antiparasitic drug discovery.

## 1. Introduction

Although epigenetic research has historically concentrated on cancer, metabolic, and neurodegenerative disorders, infectious diseases now represent a mechanistically distinct landscape in which epigenetic plasticity critically governs host–pathogen dynamics and infection outcome [1]. Several pathogens have been shown to actively remodel host epigenetic landscapes, including DNA methylation and histone modification patterns, leading to profound changes in immune cell function and inflammatory responses [1]. Importantly, such epigenetic reprogramming not only shapes host immunity but also supports pathogen persistence, virulence, and immune evasion, as demonstrated for intracellular bacterial pathogens [2].

In parallel, the emergence of targeted protein degradation strategies challenges the occupancy-driven paradigm that dominates current anti-infective drug discovery [3]. Platforms such as Lysosome Targeting Chimeras (LYTACs) [3], Autophagy Targeting Chimeras (AUTACs), and Autophagosome–Tethering Compounds (ATTECs) [4] enable the selective elimination of disease-relevant proteins through lysosomal or autophagic pathways, rather than classical functional inhibition. Foundational studies on LYTACs, AUTACs, and ATTECs demonstrate that modulation of protein homeostasis can expand the druggable space beyond conventional enzymatic targets [3].

From a conceptual standpoint, the convergence of epigenetic modulation and TPD raises a mechanistic question: if epigenetic regulators function within dynamic multiprotein assemblies, is catalytic inhibition sufficient to rewire infection-associated transcriptional states? By combining approaches that reshape host epigenetic responses with technologies capable of selectively degrading pathogen or host factors, this integrated strategy is conceptually attractive for infectious diseases. This rationale is relevant for tuberculosis, viral infections, antimicrobial-resistant bacteria, and neglected diseases, in which infection, virulence, persistence, and immune evasion often depend on regulatory protein networks.

However, several requirements must be met for this concept to become therapeutically feasible. Relevant regulatory proteins must be accessible within infected cellular compartments and productively engaged by compatible degradation machineries. In parallel, degrader chemotypes must combine permeability, metabolic stability, tissue exposure, and activity against intracellular pathogens. These requirements are particularly challenging in neglected diseases, where the number of validated targets remains limited [2,3].

Here, we discuss the intersection between epigenetic regulation and targeted protein degradation in infectious diseases, highlighting recent advances while examining the mechanistic and translational barriers that still limit their implementation in anti-infective drug discovery.

## 2. Epigenetics

In essence, epigenetics refers to heritable changes in gene expression that occur without alterations in the underlying DNA sequence and enable distinct phenotypic outcomes in response to developmental and environmental cues. The concept was first introduced in the 1940s by Conrad Waddington, who defined epigenetics as the bridge between genotype and phenotype [5,6].

Today, these modifications are recognised as central regulators of adaptive transcriptional responses and include processes such as DNA methylation, post-translational histone modifications, and regulation mediated by non-coding RNAs and microRNAs [7]. At the molecular level, these processes are governed by coordinated actions of epigenetic regulators commonly classified as writers, erasers, and readers, which, respectively, establish, remove, and interpret epigenetic marks to control chromatin organisation and gene expression [6,8].

Epigenetic mechanisms can act at two complementary levels: in the host, by shaping immune activation, tolerance, and memory, and in the pathogen, by controlling chromatin dynamics, phenotypic plasticity, and adaptation to hostile environments [8]. For example, viruses may modify host genome structure to make it more accessible for replication, whereas host cells can respond by silencing the integrated viral genome through methylation, thereby inhibiting viral gene expression [9,10]. This dual perspective highlights epigenetics as a valuable framework for understanding host–pathogen interactions and identifying novel therapeutic opportunities, particularly in infectious and neglected diseases [9].

These regulatory effects are mediated through distinct and reversible mechanisms, including DNA methylation, histone modifications, chromatin remodelling, and gene silencing mediated by non-coding RNAs [11,12,13]. Among these mechanisms, DNA methylation represents one of the most extensively studied in host–pathogen interactions [11,14].

In mammalian cells, DNA methylation is now understood as a dynamic process, with methylation patterns varying in a cell-type-specific manner and in response to environmental or pathogenic stimuli [8]. This mechanism represses genomic elements that should remain silent under specific conditions, while promoter regions of actively transcribed genes are often relatively unmethylated.

At the molecular level, DNA methylation in mammalian cells occurs predominantly at the fifth carbon of cytosine residues (5-methylcytosine), preferentially within CpG dinucleotides, which are symmetrically distributed across complementary DNA strands. This process is primarily coordinated by the DNA methyltransferase (DNMT) family, with DNMT3A and DNMT3B responsible for de novo methylation, whereas DNMT1 maintains established methylation patterns during DNA replication [8,15].

Transcriptional plasticity enables rapid and finely tuned adjustments in gene expression, allowing cells to respond to environmental and biological cues. This flexibility is particularly relevant in immune responses, where timely gene modulation is essential for host defense [7]. During host–pathogen interactions, both the host and the invading pathogen undergo extensive transcriptional reprogramming, and the molecular interplay between these adaptive responses can critically influence infection outcomes [16].

**Figure 1:**
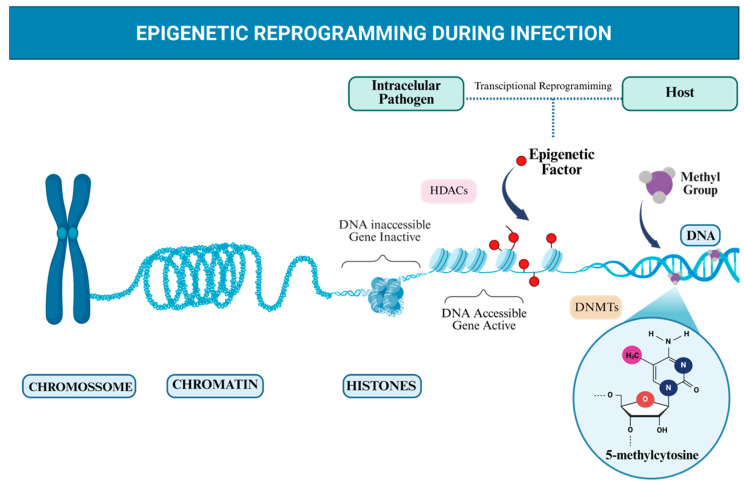
During infection, host–pathogen interactions reshape epigenetic regulation without altering the DNA sequence. Chromosomal DNA is hierarchically organised into chromatin and nucleosomes, where histone modifications regulate chromatin accessibility and transcriptional activity. At the DNA level, cytosine methylation at CpG sites—established by DNMT3A/3B and maintained by DNMT1 during replication—contributes to stable gene repression. Together, these reversible mechanisms enable transcriptional plasticity, allowing immune cells to integrate pathogen-derived signals and modulate gene expression programmes that influence infection outcome.

In macrophages, epigenetic modifications operate as environmental sensors that integrate pathogen-derived signals, inflammatory cues, and metabolic changes into coordinated transcriptional programmes. Through reversible mechanisms such as histone acetylation and methylation, these cells adjust gene expression profiles that govern polarisation states, antimicrobial effector functions, and inflammatory responses. This adaptive epigenetic plasticity is increasingly recognised as a critical determinant of infection outcome, shaping the balance between pathogen clearance and persistence [9,16,17]. These regulatory processes are schematically summarised in Figure 1.

Beyond transcriptional regulation, the functional impact of many epigenetic regulators depends on their abundance, stability, and dynamic assembly within multiprotein complexes. This structural interdependence challenges the sufficiency of occupancy-driven inhibition and provides a mechanistic rationale for targeted protein degradation strategies that eliminate, rather than transiently inhibit, regulatory components.

## 3. Targeting Protein Degradation Platforms

Traditional pharmacological approaches rely on sustained target occupancy to inhibit protein function. Although effective for many enzymes and receptors, this strategy can be limited when applied to multifunctional or highly adaptive proteins, particularly those whose biological roles extend beyond catalysis. In such cases, inhibition of catalytic activity may be insufficient, as structural interactions, scaffolding functions, regulatory roles, and compensatory mechanisms can preserve cellular phenotypes despite enzymatic blockade [18,19]. These challenges have driven the development of alternative pharmacological strategies aimed at selectively eliminating target proteins rather than merely modulating their activity.

Within this context, TPD has emerged as an event-driven pharmacological approach in which transient engagement of a target protein is sufficient to promote its removal from the cellular proteome. In contrast to occupancy-based inhibition, degradation-based strategies decouple target engagement from functional persistence, allowing sustained functional effects even after the degrading agent dissociates. Importantly, degradation removes the target protein as a functional entity, irrespective of the specific nature of its individual activities. This feature is particularly relevant for proteins whose biological function depends on abundance, complex assembly, or subcellular localisation rather than on catalytic activity alone.

The first broadly established TPD platform exploits the ubiquitin–proteasome system and is exemplified by PROTACs. These heterobifunctional molecules simultaneously engage a protein of interest and an E3 ubiquitin ligase, thereby forming a ternary complex that triggers polyubiquitination and subsequent degradation of the target by the 26S proteasome. Because PROTACs act catalytically, a single molecule can induce the degradation of multiple copies of the target protein, reinforcing the event-driven nature of this strategy.

Despite their conceptual and translational impact, PROTACs are inherently constrained by their reliance on the proteasome, which primarily processes soluble cytosolic and nuclear proteins. In addition, efficient degradation depends on suitable E3 ligase expression and accessibility, while the increased molecular complexity and physicochemical constraints of PROTACs may limit cellular permeability [20]. Together, these factors indicate that proteasome-dependent degradation is not universally applicable and underscore the need for complementary degradation pathways.

To broaden the range of targetable proteins, lysosome-dependent degradation platforms have been developed to exploit endogenous mechanisms of cellular trafficking, endocytosis, and quality control. These strategies redirect target proteins to lysosomal compartments, enabling degradation independently of the proteasome. LYTACs were specifically designed to target proteins inaccessible to the ubiquitin–proteasome system, including extracellular and plasma membrane-associated proteins. By linking a target-binding moiety to ligands recognised by lysosomal trafficking receptors, such as the cation-independent mannose-6-phosphate receptor, LYTACs promote receptor-mediated endocytosis followed by lysosomal degradation of the bound protein. This approach extends the scope of targeted protein degradation beyond intracellular proteins typically addressed by proteasome-based strategies [21].

Autophagy-based degradation strategies have also emerged as an alternative for targets that are inefficiently processed by the proteasome or that require more flexible disposal mechanisms. AUTACs harness the autophagy machinery by introducing molecular signals that mimic cellular damage or stress cues [20,22]. These signals promote autophagy-associated ubiquitination signatures and recruitment of selective autophagy receptors such as p62/SQSTM1. As a result, target proteins or larger subcellular structures are selectively sequestered into autophagosomes, which subsequently fuse with lysosomes, enabling degradation. By leveraging the intrinsic adaptability of the autophagic pathway, AUTACs allow the elimination of targets that escape strict proteasomal control.

**Figure 2:**
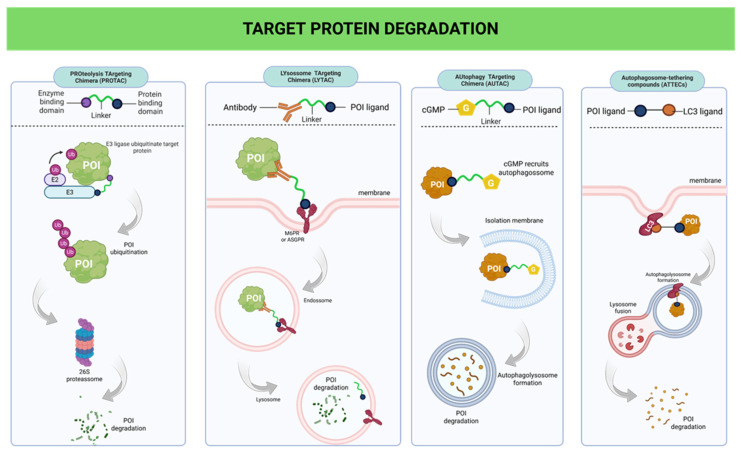
TPD platforms and their distinct proteolytic routes. Schematic representation of major degradation strategies, including PROTACs, LYTACs, AUTACs, and ATTECs. PROTACs recruit an E3 ubiquitin ligase to the POI, promoting ubiquitination and subsequent proteasomal degradation. LYTACs redirect extracellular or membrane-associated targets to lysosomal degradation via receptor-mediated endocytosis. AUTACs induce selective autophagic degradation through tagging mechanisms that trigger cargo recognition and autophagosome formation. ATTECs tether target proteins to LC3, facilitating their sequestration into autophagosomes and lysosomal clearance. Collectively, these approaches expand the druggable proteome beyond traditional occupancy-driven inhibition.

A conceptually distinct strategy is represented by ATTECs, which induce protein degradation independently of ubiquitination. Rather than relying on degradation signals, ATTECs directly link the target protein to structural components of the autophagosome, such as LC3, thereby enabling its selective incorporation into autophagic vesicles [4]. Bypassing the ubiquitin–proteasome system, ATTECs enable protein degradation through autophagy-based mechanisms that are mechanistically distinct from proteasome-dependent approaches. Figure 2 summarizes the TPD strategies and their distinct proteolytic pathways.

Collectively, these platforms illustrate that targeted protein degradation constitutes a modular pharmacological framework rather than a single technology, with each strategy leveraging distinct cellular clearance pathways. The choice of platform is therefore dictated not only by target accessibility, but also by subcellular localisation, structural context, and dependency on specific proteostatic networks. However, extending these strategies to infectious diseases requires consideration of pathogen-specific proteostasis systems, intracellular niche accessibility, and host–pathogen proteome crosstalk.

Despite the advantages of TPD approaches, several limitations remain. First, the efficiency of PROTAC-mediated degradation depends not only on target binding but also on the formation and stability of the ternary complex involving the protein of interest and the recruited E3 ligase, which must be sufficiently stable to enable effective ubiquitination. Cooperative interactions within this complex can enhance ubiquitination efficiency even when binary affinities are moderate. Conversely, at high concentrations, PROTACs may exhibit the so-called “hook effect,” in which excessive formation of binary complexes limits productive assembly of ternary complexes [23].

In addition, PROTACs often present pharmacokinetic challenges, including high molecular weight, increased polarity, and reduced membrane permeability, which may compromise their bioavailability and translational potential [24]. To provide a structured overview, this section discusses the key advantages and limitations of TPD strategies and summarises them in Table 1.

Although TPD has emerged as a transformative strategy in chemical biology, its application to infectious diseases remains constrained by several unresolved factors, particularly in parasitic diseases. These include limited knowledge of exploitable degradation machineries in pathogens, uncertainty regarding suitable ligases or alternative proteolytic systems, restricted access to targets within infected cellular compartments, and the challenge of designing degrader chemotypes with sufficient permeability, stability, and tissue exposure. Together, these limitations are especially relevant to intracellular and neglected infections, where both target validation and translational development remain underdeveloped.

## 4. TPD in Infectious Disease Applications

Epigenetic regulation contributes to pathogen adaptability and persistence during infection, particularly under host-imposed and therapeutic pressures. In bacteria and other infectious agents, DNA methylation and chromatin-like regulatory systems, driven by specialised DNA-modifying enzymes, modulate transcriptional programmes that control virulence, stress responses, metabolic adaptation, and antimicrobial tolerance [16,17].

Concomitantly, viruses and intracellular bacteria reshape host chromatin architecture to sustain replication and persistence, influencing cell-cycle regulation, apoptosis, and immune responses [17]. Because these processes depend on regulatory proteins that coordinate transcriptional programmes and the assembly of multiprotein complexes, strategies targeting protein stability have attracted increasing attention. In this context, targeted protein degradation emerges as a mechanistically attractive approach to disrupt infection-associated epigenetic reprogramming by eliminating key regulatory nodes rather than transiently inhibiting catalytic activity. This raises a central question: across different infectious diseases, which regulatory vulnerabilities are realistically addressable by targeted protein degradation, and what biological constraints limit their therapeutic translation?

### 4.1. Antimicrobial-Resistant Bacteria

AMR now represents a systemic failure of conventional antibiotic paradigms, threatening both clinical practice and global health security [25]. According to recent WHO reports, approximately one in six common bacterial infections is now resistant to standard treatments, undermining the effectiveness of modern medicine [26]. As antibiotic efficacy declines, routine medical procedures, including surgery, chemotherapy, and organ transplantation, become increasingly hazardous due to the risk of untreatable infections [27]. This scenario is driven not only by accelerated pathogen evolution but also by a stagnant pharmaceutical pipeline that renders antimicrobial development economically unattractive [28]. In the absence of novel intervention strategies, AMR may cause millions of deaths and substantial global economic losses [29].

Beyond classical resistance mechanisms, bacterial pathogenicity is reinforced by virulence factors that enhance immune evasion, tissue invasion, and persistence [30,31]. Many of these virulence determinants are secreted proteins or surface-exposed effectors that contribute to tissue damage and persistence. Several of these factors have been experimentally characterised and play critical roles in pathogenicity. Such extracellular or membrane-associated proteins may, in principle, be accessible to emerging extracellular TPD strategies such as LYTACs.

To date, however, this possibility remains hypothetical, as no study has yet demonstrated LYTAC-mediated degradation in bacterial systems. While the accessibility of these virulence factors suggests a potential opportunity for degradation-based approaches, this strategy still requires experimental validation in bacterial infection models. Rather than exerting direct bactericidal pressure, depletion of virulence factors could attenuate pathogenic fitness while minimising selective pressure for resistance.

For example, in *Acinetobacter baumannii*, the secreted protease PKF promotes complement evasion and serum resistance [32,33], while CpaA functions as a zinc-dependent glycoprotease contributing to host tissue damage [34,35]. Similarly, extracellular toxins in ESBL-producing *Escherichia coli*, including HlyA, CNF1, and Sat, disrupt epithelial integrity and immune signalling [36,37]. In *Pseudomonas aeruginosa*, LasB, AprA, Exotoxin A, and the biofilm-associated adhesin CdrA promote tissue destruction, immune evasion, and biofilm stability [38,39]. Likewise, *Staphylococcus aureus* secretes cytolysins and leukocidins such as Hla and PVL that directly damage host cells and exacerbate inflammation [40]. Collectively, these extracellular effectors illustrate how protein-level depletion strategies could complement conventional antimicrobials by targeting pathogenic mechanisms rather than bacterial viability per se.

Importantly, antimicrobial resistance cannot be fully explained by genetic mutations and horizontal gene transfer alone. Reversible epigenetic regulation has emerged as an additional layer modulating antimicrobial tolerance and persistence [41]. DNA methyltransferases, including Dam, Dcm, and HsdM, as well as phase-variable methyltransferases and nucleoid-associated proteins such as H-NS and HU, modulate the expression of efflux pumps, stress response pathways, conjugative machinery, and biofilm-related genes. These mechanisms enable heritable yet reversible phenotypic variation without alterations in DNA sequence, thereby facilitating rapid adaptation to antibiotic pressure [41].

Recent studies further support the relevance of epigenetic remodelling in resistant pathogens. Transcriptome-methylome analyses in *A. baumannii* revealed widespread differential m5C methylation patterns associated with resistance phenotypes, implicating cytosine and adenine methyltransferases as higher-order regulatory nodes [42]. In *E. coli*, global methylation shifts under subinhibitory antibiotic exposure highlight the role of Dam, Dcm, and HsdM in transient adaptive resistance [43]. Although early studies identified small-molecule Dam inhibitors [44], pharmacological targeting of bacterial methyltransferases remains largely unexplored. Similarly, genome-wide m6A mapping in clinical *P. aeruginosa* isolates identified methylation-associated loci linked to stress adaptation and persistence [45].

Together, these observations suggest that epigenetic enzymes themselves constitute attractive protein-level targets. Selective degradation of bacterial methyltransferases or other epigenetic regulators may interfere with adaptive resistance programmes at their regulatory core, potentially resensitising pathogens without directly imposing bactericidal pressure. In principle, such an approach may decouple survival from adaptability, targeting the regulatory architecture of resistance rather than bacterial viability itself.

Nevertheless, it remains unclear whether degradation of epigenetic regulators would produce durable resensitisation or whether bacterial regulatory plasticity could still enable compensatory adaptive responses.

### 4.2. Tuberculosis and Other Mycobacterial Infections

Tuberculosis (TB) remains the leading cause of death from a single infectious agent worldwide, with 8.2 million new cases and 1.25 million deaths reported in 2023 [46]. The emergence of multidrug-resistant tuberculosis (MDR-TB) and extensively drug-resistant tuberculosis (XDR-TB), together with the ability of *Mycobacterium tuberculosis* (Mtb) to persist in a dormant state within macrophages, poses major obstacles to current therapeutic strategies. Following inhalation, Mtb is phagocytosed by macrophages but survives intracellularly by evading xenophagy, a specialised form of autophagy that eliminates intracellular pathogens [47].

Although xenophagy relies on the canonical AMPK-mTOR-ULK1 signalling axis and LC3-dependent autophagosome formation [48,49,50], Mtb actively subverts this pathway. Secreted effectors such as SapM inhibit RAB7-dependent autophagosome maturation [51,52], PtpA disrupts vacuolar ATPase activity and autophagosome acidification [53], and EsxA-mediated membrane damage, together with the EsxG–EsxH heterodimer, interferes with membrane repair processes [54]. Enhanced intracellular survival (EIS) proteins further suppress xenophagic clearance through acetylation of host DUSP16, thereby dampening inflammatory signalling and promoting intracellular persistence [55]. These protein effectors represent attractive candidates for protein-level intervention, as their depletion could restore host antimicrobial pathways rather than directly targeting bacterial viability.

Increasing evidence indicates that Mtb actively manipulates host epigenetic machinery to suppress antimicrobial responses. Recruitment of HDAC1 and other chromatin-modifying enzymes to promoters of pro-inflammatory genes promotes transcriptional silencing, contributing to intracellular persistence. Pharmacological HDAC inhibition restores antimicrobial peptide expression and enhances oxidative defence pathways, supporting host-directed therapeutic strategies [56].

**Figure 3:**
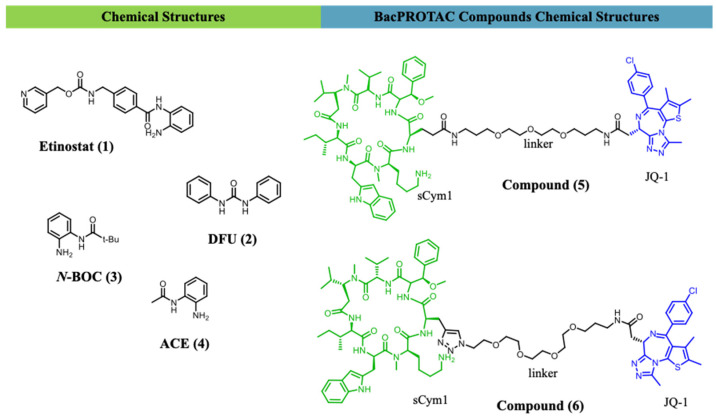
Chemical structures of classic HDACi and BacPROTAC compounds tested against *M. tuberculosis* and *M. smegmatis*, respectively. JQ1 and the cyclomarin derivative sCym1 are depicted in blue and green, respectively.

Rodríguez-Carlos et al. (2023) demonstrated that entinostat (**1**), DFU (**2**), N-BOC (**3**), and ACE (**4**) (Figure 3) reduced intracellular H_37_Rv burden in infected macrophages and pneumocytes without exerting direct bactericidal effects, while enhancing the expression of antimicrobial peptides (LL-37, HBD-2, β-defensin) and oxidative mediators such as SOD3 and iNOS [57]. These findings raise the possibility that targeted degradation of selected epigenetic repressors may extend the host-directed effects observed with enzymatic inhibition, although this concept remains to be validated in tuberculosis models [57].

Selective depletion of host epigenetic repressors or Mtb-derived effector proteins could, in principle, enable more durable reprogramming of antimicrobial transcriptional networks [57]. Surface-exposed or cytosol-accessible mycobacterial proteins, such as the antigen 85 complex involved in cell wall biosynthesis [58,59], and LprG, a virulence-associated lipoprotein that interacts with TLR2 and contributes to immune evasion [60], represent potential candidates for AUTAC- or ATTEC-based strategies, although this remains hypothetical.

Given that many Mtb proteins lack well-defined hydrophobic pockets suitable for conventional small-molecule inhibition [61], degradation-based approaches may enable the targeting of otherwise inaccessible proteins [62,63].

The interplay between mycobacterial effectors, host epigenetic reprogramming, and autophagic evasion highlights a multilayered regulatory network in which protein levels influence infection outcome. TPD provides a strategy to intervene at this level, either by eliminating bacterial virulence determinants or by modulating host epigenetic regulators, thereby rebalancing host–pathogen interactions in drug-resistant TB.

While host-directed degradation aims to restore antimicrobial pathways, pathogen-directed degradation offers the possibility of directly dismantling bacterial proteostatic networks. In this context, pathogen-intrinsic degradation pathways can also be harnessed to selectively eliminate essential mycobacterial proteins.

BacPROTACs are dual-function synthetic adaptors designed to bridge a specific target protein with the bacterial ClpC-ClpP (ClpCP) protease system. These molecules consist of a targeting ligand linked to a recruiter group that engages the *N*-terminal domain of ClpC, effectively mimicking endogenous degradation cues such as phospho-arginine. This simultaneous binding triggers a conformational shift in ClpC, converting it from a dormant state into an active, higher-order oligomer. Once activated, the ClpC engine uses ATP hydrolysis to mechanically unfold the captured protein and deliver it to the ClpP proteolytic core for destruction [64].

A study by Morreale and collaborators (2022) [64] demonstrated that BacPROTACs can selectively degrade the POI. The *Mycobacterium smegmatis* (Msm) strain was engineered to express the BRDTBD1 protein, and BacPROTACs were synthesised using JQ1 as the target ligand and cyclomarin derivatives as ClpC ligands (Figure 3).

Cell cultures incubated with the compounds (**5**) and (**6**) exhibited a significant reduction in BRDTBD1 concentration compared to DMSO. After 30 min, cultures treated with compound (**5**) exhibited a BRDTBD1 reduction of approximately 50% at 10 µM and over 50% at 100 µM. After 2 h of incubation with compound (**5**), this reduction was maintained, whereas incubation with compound (**6**) at a concentration of 20 µM for the same duration resulted in a 25% reduction. In addition, compound (**6**) bound to ClpC with high affinity (K = 0.2 µM), suggesting a strong interaction with the Msm degradation machinery [64].

Although BacPROTACs provide an important proof of concept for TPD in mycobacterial systems, current evidence remains largely preclinical and is still limited by species-dependent responses, reliance on engineered or non-native substrates in some settings, and unresolved pharmacokinetic liabilities [65,66]. At the same time, recent studies showing activity against intracellular and quiescent Mtb support the view that this strategy has moved beyond a purely conceptual stage, although its broader translational relevance remains to be established. Importantly, differences between Msm and Mtb, including growth rate, metabolism, virulence, and possibly the expression of alternative Clp ATPases, such as ClpC2 and/or ClpC3, may influence susceptibility to BacPROTAC-mediated degradation. These differences limit direct extrapolation from Msm to infection-relevant Mtb settings.

Despite these conceptual advantages, important limitations remain, particularly for applications targeting Mtb or Msm, as illustrated by the studies discussed above. This is largely because the available literature is still predominantly focused on HDACi and host-directed approaches, without demonstrating protein degradation or establishing whether enzymatic inhibition alone is sufficient to promote durable regulatory remodelling in in vivo models. In addition, modulation of host epigenetic regulators may carry toxicity risks, given their central role in immune regulation.

The application of AUTAC- and ATTEC-based strategies in Mtb remains hypothetical, as no experimental studies have yet validated these approaches in Mtb or Msm models. Together, these limitations highlight unresolved translational challenges, including intracellular delivery to infected macrophages, target accessibility at the host–pathogen interface, and demonstration of efficacy in clinically relevant tuberculosis models. The compounds described in this section are summarised in Table 2.

### 4.3. Viral Infections

Viral infections pose unique pharmacological challenges, as their replication and persistence are dependent on host cellular machinery. Chronic viral infections such as human immunodeficiency virus (HIV) affect nearly 40 million people worldwide [67], while hepatitis B and C affect more than 300 million individuals and are responsible for over one million deaths annually [68]. In addition, highly prevalent RNA viruses, including dengue virus [69] and influenza virus [70], cause hundreds of millions of infections each year, underscoring the persistent need for new antiviral strategies. In this context, protein degradation platforms have attracted attention due to their expanding applicability across different therapeutic areas and drug discovery programmes. Unlike bacterial pathogens, viruses rely almost entirely on host transcriptional and proteostatic systems, making host–virus protein interfaces particularly attractive yet complex therapeutic targets.

Among viral chronic infections, HIV-1 represents a model of chromatin-mediated viral persistence. Although antiretroviral therapy (ART) effectively suppresses viraemia, it fails to eradicate infection because a stable latent reservoir is established within long-lived CD4+ T cells [71,72,73,74,75,76]. Latency-reversing agents (LRAs) have been explored as part of the “shock-and-kill” strategy to eliminate this reservoir by reactivating viral gene expression and exposing infected cells to immune-mediated clearance. However, LRAs induce only partial proviral reactivation and have not consistently reduced reservoir size. Long-lived latently infected CD4+ T cells, therefore, remain the principal barrier to HIV-1 eradication [74]. These limitations underscore the difficulty of reversing established latency and support greater interest in strategies that interfere with latency establishment [75].

At the molecular level, latency establishment is linked to the transition of activated CD4+ T cells into a resting state, accompanied by reduced availability of transcription-promoting host factors, including NF-*κ*B, P-TEFb, and RNA polymerase II. This process is reinforced by repressive epigenetic marks, including H3K9me3 and H3K27me3, and by integration into transcriptionally restrictive chromatin regions. These events are regulated by host chromatin-modifying complexes, including histone acetyltransferases (HATs), HDACs, histone methyltransferases (HMTs), and lysine demethylases (KDMs) [77,78,79]. Among these regulators, HDAC3 has emerged as a relevant contributor to HIV-1 latency control [75,80].

Several epigenetic targets have previously been explored as latency-reversing nodes in HIV-1 infection, including HDACs, histone methyltransferases such as G9a, EZH2 and SMYD2, and bromodomain proteins such as BRD4, reinforcing chromatin regulation as a central axis in viral persistence [75,76,77,78,79]. Among these, HDAC inhibitors have been the most extensively investigated as LRAs. In cellular and ex vivo models, compounds such as vorinostat, givinostat, belinostat, and panobinostat induced proviral reactivation, exhibiting distinct potency profiles; the overall activity trend was reported as panobinostat > givinostat ≈ belinostat > vorinostat > valproic acid [73,75]. In this context, selective degraders provide an opportunity to test whether removing individual epigenetic regulators offers mechanistic or functional advantages over enzymatic inhibition alone.

**Figure 4:**
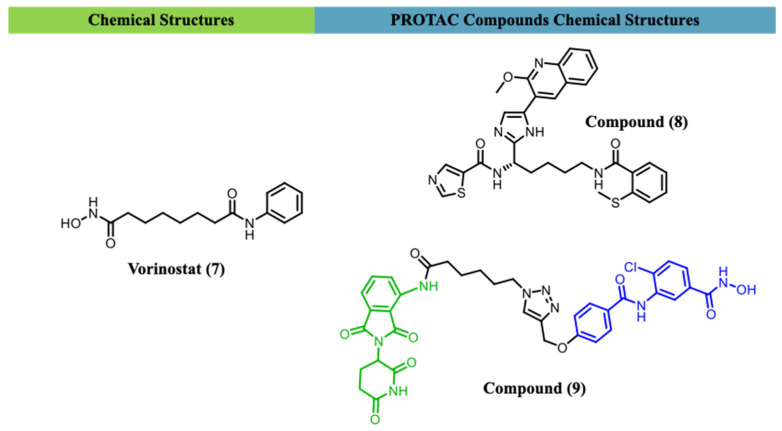
Chemical structures of vorinostat and PROTAC compounds tested in HIV-infected and IAV-infected cells, respectively. In the PROTAC structure (**9**), the HDAC inhibitor moiety is highlighted in blue, whereas the E3 ligase-recruiting ligand is highlighted in green.

The HDAC3-selective PROTAC compound (**8**) (Figure 4) provided a chemical tool to interrogate the role of selective degradation in this process [78]. Peterson et al. (2023) showed that targeting HDAC1, HDAC2, or HDAC3 was sufficient to impair latency establishment, whereas reversal of established latency required broader disruption of all three isoforms [75]. In primary CD4+ T cells, 40 nM compound (**8**) induced robust and relatively selective HDAC3 degradation (approximately 77% reduction in protein levels) but did not trigger latency reversal. At concentrations ≥ 100 nM, concomitant degradation of HDAC1 and HDAC2 was observed, coinciding with latency reversal and indicating that selective HDAC3 depletion alone is insufficient to sustain proviral reactivation [75,80].

In prevention assays, three weeks of treatment with 40 nM compound (**8**) reduced latency establishment to levels comparable to 250 nM vorinostat, although prolonged exposure compromised selectivity [75,80]. Mechanistically, early HDAC inhibition increased H3K9ac and reduced H3K9me3 at the viral LTR, supporting histone deacetylation as an initiating event in proviral silencing. Collectively, these findings support HDAC-directed degradation primarily as a strategy to interfere with latency establishment rather than to reverse established latency [75].

Unlike conventional shock-and-kill approaches, which rely on transient proviral reactivation followed by immune-mediated clearance, targeted protein degradation enables direct and sustained removal of host or viral proteins that regulate latency, persistence, or replication. This expands antiviral intervention beyond transcriptional reactivation alone.

Beyond HDAC-directed latency-reversing strategies, recent work has also explored bromodomain-targeting degraders in HIV-1 latency models. BRD4-targeting PROTACs incorporating HIV-1 Vpr-derived peptides as E3 ligase-binding components were shown to recruit the DCAF1–DDB1–Cul4A ubiquitin ligase complex, broadening ligase-recruitment strategies beyond canonical CRBN- or VHL-based designs. However, these BRD4 degraders were less effective than the parent inhibitor JQ1 as latency-reversing agents, although their lower cytotoxicity allowed higher concentrations to be used and still produced measurable LRA activity [77,78,79].

A 2025 study focused on influenza A virus (IAV) reported the development of compound (**9**) as a selective PROTAC degrader targeting HDAC6. Compound (**9**) incorporates an HDAC6 inhibitory warhead connected through a polyethylene glycol (PEG) linker to a cereblon (CRBN)-recruiting ligand derived from lenalidomide (Figure 4). Proof-of-concept experiments demonstrated effective HDAC6 degradation at submicromolar concentrations (0.1–0.5 µM), highlighting the feasibility of selectively depleting this epigenetic regulator through a heterobifunctional strategy [81].

HDAC6 represents an attractive antiviral target, as it participates in the dissociation of the viral ribonucleoprotein (vRNP) complex from the matrix protein M1 and facilitates capsid disassembly by recruiting molecular motor proteins that promote vRNP transport into the nucleus [82]. Notably, the absence of HDAC6 does not critically compromise host cell viability, supporting its suitability as a target within degradation-based therapeutic strategies [81].

Collectively, these examples illustrate how TPD may broaden antiviral strategies by enabling the selective elimination of host factors or viral proteins involved in latency, replication, or intracellular trafficking. TPD provides a mechanistically distinct approach that may help overcome limitations associated with conventional inhibition.

Beyond chromatin-mediated regulation, viral epitranscriptomic mechanisms may also expand the antiviral target space. For example, in SARS-CoV-2, nsp14 and nsp16 mediate 5′ RNA cap methylation, thereby promoting viral translation and dampening innate immune sensing, which supports these enzymes as plausible antiviral targets [83]. Despite growing interest, the integration of degradation platforms with viral persistence mechanisms remains in its early stages, representing a promising direction for antiviral medicinal chemistry [84]. The compounds described in this section are summarised in Table 3.

Important limitations remain. The studies described predominantly focus on host-directed strategies and do not demonstrate whether selective degradation of a single epigenetic regulator is sufficient to achieve durable effects. In the context of HIV-1, the results indicate that modulation of multiple HDAC isoforms is required, as selective degradation of HDAC3 alone was not sufficient to reverse established latency. In addition, at higher concentrations, a loss of selectivity was observed, associated with the concomitant degradation of HDAC1 and HDAC2. Similar observations were found for BRD4 modulation, in which PROTACs were less active than the direct inhibitor JQ1. Furthermore, the available evidence is restricted to cellular models, and the impact of these strategies in more complex systems has not been demonstrated.

In the case of IAV, although HDAC6 degradation represents a proof of concept, its effect on the overall dynamics of infection was not explored. Overall, these studies highlight persistent challenges related to selectivity, the number of targets involved, and the lack of validation in models that more closely reflect the physiological context.

## 5. TPD Applications in Parasitic Neglected Diseases

Neglected tropical diseases still suffer from major therapeutic limitations, including toxic, prolonged, or insufficiently effective treatments, as well as a limited number of well-validated molecular targets. In this context, the application of targeted protein degradation may appear premature. However, growing evidence indicates that epigenetic regulators play important roles in parasite differentiation, virulence, host adaptation, and transmission, suggesting that regulatory proteins may represent mechanistically relevant intervention points even in therapeutically underdeveloped systems.

Because direct evidence for targeted protein degradation in neglected parasitic diseases remains scarce, and only a limited fraction of these studies involves epigenetic regulators, the discussion in this section necessarily draws more extensively on the epigenetics literature to identify biologically relevant targets and to define the mechanistic context in which TPD may become feasible.

In protozoan pathogens, chromatin-associated complexes govern stage conversion, immune evasion, and stress responses. HDAC and bromodomain inhibitors have already provided proof-of-concept activity, indicating that epigenetic regulation is pharmacologically tractable at least at the level of enzymatic inhibition [3,85].

We therefore focus on four major protozoan diseases, namely Chagas disease, human African trypanosomiasis, leishmaniasis, and malaria, in which epigenetic regulation plays a central role in stage differentiation, immune evasion, and transmission, providing a conceptual framework to evaluate the translational potential of targeted protein degradation in neglected infections.

The translation of TPD strategies to infectious diseases, particularly parasitic infections, remains challenging. The intracellular niche occupied by many parasites, combined with their complex and resilient biology, limits the feasibility of using host E3 ligases to achieve selective degradation of parasite proteins within infection-relevant cellular compartments. Although protozoan parasites possess ubiquitination machinery and functionally characterised E3 ligases, including HECT-type ligases and cullin-RING complexes in *Leishmania* and *Plasmodium*, the repertoire of parasite E3 ligases remains poorly characterised from a chemical biology perspective, and ligandable recruiters analogous to CRBN or VHL have not been established. Alternative approaches, such as ATTEC- or LYTAC-based platforms, may represent more plausible strategies, although their applicability to intracellular parasitic targets remains largely unexplored.

### 5.1. Chagas Disease (Trypanosoma cruzi)

Chagas disease, also known as American trypanosomiasis, is a potentially life-threatening infection caused by the protozoan *T. cruzi*. Transmission occurs primarily through contact with the faeces of infected triatomine insects, although congenital, transfusional, oral, and transplant-associated routes have also been reported. More than seven million people are currently infected worldwide, predominantly in endemic regions of Latin America, and over 100 million remain at risk [86]. Delayed diagnosis and treatment of chronic infection may lead to progressive cardiac and digestive complications, increasing morbidity and mortality. Although curative therapy is effective in the early stages, treatment options in the chronic phase remain limited and may be associated with adverse effects, underscoring the need for new therapeutic strategies grounded in a mechanistic understanding of parasite biology [87].

Gene expression in *T. cruzi* is largely uncoupled from classical transcriptional control, placing chromatin organisation and post-translational histone modifications at the centre of parasite regulation. Throughout its life cycle, developmental transitions and infective competence depend on mechanisms that coordinate broad transcriptional responses in the absence of gene-specific promoters [88].

Within this biological context, histone acetylation has been shown to function as an active regulatory layer in *T. cruzi* [89]. By comparing the effects of histone deacetylase modulation across different parasite stages, the study established a direct relationship between chromatin acetylation states and parasite development. Inhibition of class I/II HDACs with trichostatin A, together with modulation of sirtuins using sirtinol or resveratrol, allowed the comparison of hyperacetylated and hypoacetylated chromatin states and their biological consequences.

These interventions generated distinct acetylation profiles in *T. cruzi*. Broad HDAC inhibition induced global histone hyperacetylation, whereas modulation produced more restricted or opposing effects. These changes translated into stage-dependent phenotypes. HDAC inhibition impaired metacyclogenesis while increasing trypomastigote infectivity, whereas resveratrol reduced epimastigote proliferation and limited intracellular amastigote replication. Together, these findings indicate that acetylation-dependent regulation in *T. cruzi* is tightly linked to developmental stage and parasite fitness [89].

The effects of acetylation were not limited to global phenotypes. The perturbation of histone acetylation alters the expression of chromatin-associated factors, including bromodomain-containing proteins. In particular, changes in BDF2 transcript levels provided an experimental link between global chromatin remodelling and epigenetic reader proteins. These findings suggested that, although broad modulation of acetylation affects parasite biology, selective targeting of defined chromatin-associated components may offer greater mechanistic precision [89].

Building on this premise, subsequent studies examined whether pharmacological modulation of epigenetic enzymes could be translated into selective antiparasitic activity. Di Bello et al. (2022) evaluated a panel of structurally diverse HDAC inhibitors in *T. cruzi*–infected L929 fibroblasts, combining phenotypic assays with host–cell cytotoxicity measurements [90].

**Figure 5:**
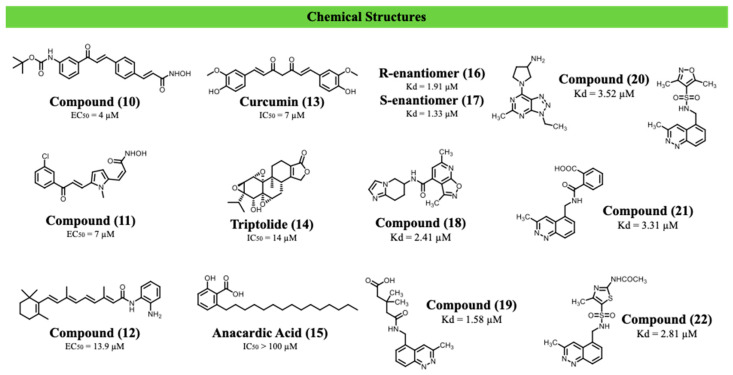
Chemical structures of epigenetic modulators targeting global acetylation processes in *T. cruzi*, including HDAC inhibitors and histone acetyltransferase inhibitors, with their reported antiparasitic activities (EC_50_ or IC_50_, µM).

Compound (**10**) displayed the highest antiparasitic potency (EC_50_ = 4 µM), but its poor selectivity (CC_50_ = 5 µM; SI = 1.25) limited its further development. In contrast, compound (**11**), which selectively inhibits HDAC6/8 while sparing nuclear HDAC1-3, achieved a more favourable balance between activity (EC_50_ = 7 µM) and toxicity (CC_50_ = 40 µM; SI = 5.7). Compound (**12**) exhibited moderate antiparasitic activity (EC_50_ = 13.9 µM) and low cytotoxicity. These results reinforce the relevance of HDACs to parasite survival while highlighting limitations associated with the high conservation of catalytic domains between parasite and host (Figure 5).

Additional insight into the role of acetylation was provided by Bortolami et al. (2025), who investigated the effects of histone acetyltransferase inhibition on parasite viability and ultrastructure [91].

Curcumin (**13**) inhibited epimastigote proliferation (IC_50_ = 7 µM) and reduced intracellular amastigote replication, accompanied by nuclear alterations, including heterochromatin decondensation and nucleolar disorganisation. Triptolide (**14**) also impaired parasite growth but exhibited substantial host–cell toxicity, whereas anacardic acid (**15**) did not significantly affect parasite proliferation despite inducing mitochondrial alterations. These findings support acetylation as a key regulator of nuclear organisation and viability in *T. cruzi*, while highlighting the limited translational potential of poorly selective epigenetic modulators (Figure 5).

Together, these results indicate that, although enzymatic inhibition affects parasite development, limited selectivity and conserved active sites constrain translational progress, supporting the exploration of alternative strategies. These studies establish epigenetic regulation as a determinant of *T. cruzi* development and highlight the limitations of nonspecific epigenetic interventions. This combination of biological relevance and pharmacological constraints has driven the search for more selective targets, particularly reader proteins that translate acetylation marks into defined regulatory outputs.

A relevant step in this direction was reported by Tavernelli et al. (2024), who evaluated the chemical tractability of the bromodomain factor TcBDF2 [92]. Using a fluorescence polarization–based high-throughput screening assay, the authors screened a 28,251-compound library against recombinant TcBDF2 and identified seven small molecules with measurable binding activity (pIC_50_ ≥ 4.5). Target engagement was confirmed by differential scanning fluorimetry, yielding dissociation constants in the low micromolar range, namely 1.91 µM (**16**), 1.33 µM (**17**), 2.41 µM (**18**), 1.58 µM (**19**), 3.52 µM (**20**), 3.31 µM (**21**), and 2.81 µM (**22**). Docking analyses indicated occupation of the acetyl-lysine recognition pocket, including interactions with conserved residues such as Asn86 and Trp92.

Despite confirmed biochemical binding, none of the compounds exhibited significant trypanocidal activity against epimastigotes or intracellular amastigotes at concentrations up to 50 µM. These results demonstrated that TcBDF2 is structurally ligandable, but identified compounds failed to demonstrate significant cellular activity, highlighting a disconnect between biochemical binding and functional efficacy (Figure 5).

In contrast, Alonso et al. (2016) demonstrated that the bromodomain-containing protein TcBDF3 has been shown to play an active regulatory role in parasite development [93]. Genetic overexpression of TcBDF3 impaired epimastigote proliferation, reduced metacyclogenesis, and decreased host–cell infectivity without evidence of generalised toxicity, consistent with a dosage-sensitive role within chromatin-associated complexes [93].

Having established its biological relevance, Alonso et al. (2024) subsequently explored the chemical tractability of TcBDF3 [94]. Using a fragment-based and dynamic combinatorial chemistry approach, the authors identified hydrazone-derived compounds and corresponding 1,3,4-oxadiazole analogues with measurable affinity for TcBDF3 and stage-dependent antiparasitic activity. Compound (**23**) emerged as the most potent TcBDF3 binder (K_d_ = 1.7 µM; IC_50_ = 2.4 µM), displaying activity against epimastigotes (EC_50_ = 23.0 µM), trypomastigotes (17.8 µM), and intracellular amastigotes (13.1 µM), while maintaining low cytotoxicity (CC_50_ = 269 µM). A5B4 showed weak affinity for TcBDF3 (K_d_ = 136 µM) and was not prioritised.

**Figure 6:**
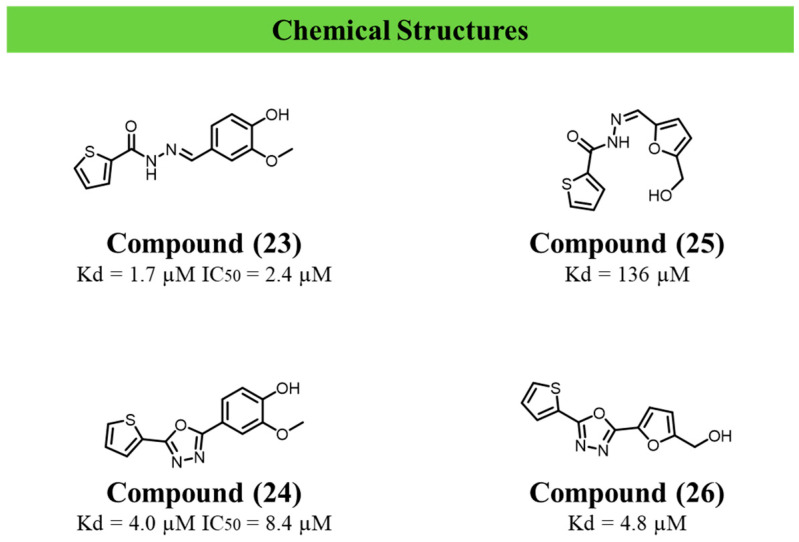
Structures and biological profiles of TcBDF3 ligands identified by fragment-based and dynamic combinatorial chemistry approaches described by Alonso et al. [94].

Structural optimisation of the hydrazone scaffold yielded oxadiazole derivatives with improved cellular potency. Compound (**24**) retained micromolar affinity for TcBDF3 (K_d_ = 4.0 µM; IC_50_ = 8.4 µM) and exhibited enhanced antiparasitic activity, particularly against trypomastigotes (EC_50_ = 1.67 µM), alongside activity against epimastigotes (8.13 µM) and amastigotes (6.9 µM). Although cytotoxicity increased relative to compound (**23**) (CC_50_ = 90.8 µM), the compound maintained a favourable selectivity profile. Compound (**26**) also showed improved binding affinity (K_d_ = 4.8 µM) and antiparasitic activity across parasite forms, while exhibiting low host–cell toxicity (CC_50_ = 315.6 µM), albeit with lower overall potency [94] (Figure 6). The compounds described in this section are summarised in Table 4.

These studies establish TcBDF3 as both a biologically relevant regulator and a chemically addressable epigenetic reader in *T. cruzi*. The observation that perturbation of TcBDF3 levels or function produces marked phenotypic effects supports strategies that act directly at the protein level, rather than relying solely on enzymatic inhibition, and naturally connects epigenetic reader biology to approaches aimed at regulated protein abundance or stability.

In this broader context, Boer and Bijlmakers (2019) examined whether components of the ubiquitin-proteasome system (UPS) in trypanosomatids could be selectively targeted without affecting the host mechanism [95]. Focusing on the ubiquitin-activating enzyme E1 (UBA1), the authors showed that *Trypanosoma brucei* expresses two catalytically active isoforms, TbUBA1a and TbUBA1b, both capable of supporting ubiquitin transfer in vitro.

Notably, the human UBA1 inhibitor TAK-243 failed to inhibit either parasite enzyme, a resistance explained by specific amino acid substitutions within the adenylation domain, including differences at the gatekeeper residue. These features are conserved in UBA1 homologues from *T. cruzi* and *Leishmania* spp., indicating that ubiquitination in trypanosomatids is essential yet structurally distinct from that in humans.

This biochemical evidence is further supported by Gini et al. (2025), who discuss the feasibility of exploiting the *T. cruzi* UPS for TPD strategies [85]. The authors highlight that the parasite UPS is fully functional but considerably less complex than its mammalian counterpart, comprising approximately 269 associated proteins compared with more than 1200 in humans. This reduced complexity suggests limited redundancy and increases sensitivity to perturbation of individual components. Within this framework, proteins such as TcBDF3, TcHsp90, TcTopoIIα, and cruzain emerge as potential candidates for degradation-based approaches, supported by existing ligandability data and clear experimental pipelines for validation.

Together, these studies indicate that the UPS in trypanosomatids is essential, structurally distinct from the human machinery, and amenable to selective chemical intervention. Combined with the demonstrated biological relevance and ligandability of epigenetic readers such as TcBDF3, these findings support the exploration of TPD strategies in Chagas disease, particularly those aimed at regulatory scaffolds and chromatin-associated complexes.

Parasites exhibit highly adaptable drug-resistance mechanisms. As reported by Atwood et al. (2005), transcriptomic and proteomic analyses of *T. cruzi* bloodstream trypomastigotes and cultured parasites revealed differential expression of more than 2000 proteins, highlighting the extensive plasticity of parasite biology [96]. This adaptive capacity enables the rewiring of metabolic and regulatory networks, allowing inhibition of a single pathway to be buffered by compensatory responses that preserve parasite survival.

Taken together, these findings indicate that the current rationale for degradation-based strategies in *T. cruzi* does not arise from broad epigenetic enzyme inhibition alone. Rather, it emerges from the convergence of three factors: the clear biological relevance of chromatin regulation, the emerging ligandability of reader proteins such as TcBDF3, and the existence of a parasite UPS that is essential yet structurally distinct from its human counterpart. At the same time, direct evidence for TPD in Chagas disease remains scarce, and most of the available literature still focuses on epigenetic modulation rather than protein degradation itself. This imbalance suggests that the field has advanced far enough to define biologically meaningful targets, but not yet far enough to establish the most suitable degradation architecture. In this context, canonical PROTAC strategies may not represent the most immediate route, particularly given the lack of chemically tractable parasite recruiters analogous to CRBN or VHL. Future progress will likely depend on identifying degradation-compatible parasite components or adapting non-canonical TPD modalities to the intracellular and highly plastic biology of *T. cruzi*.

### 5.2. Sleeping Sickness

Human African trypanosomiasis, or sleeping sickness, is a NTD caused by infection with *Trypanosoma brucei* (*T. brucei*), a flagellated protozoan transmitted through the bite of infected tsetse flies. The disease occurs mainly in sub-Saharan Africa and is caused by two epidemiologically and clinically distinct subspecies: *T. brucei gambiense***,** responsible for the majority of cases and typically associated with a slowly progressive infection, and *T. brucei rhodesiense*, which generally produces a more rapidly evolving illness [97].

Following transmission, parasites initially proliferate in blood and lymphatic tissues before, in untreated individuals, crossing the blood–brain barrier and establishing infection within the central nervous system. Neurological involvement marks the advanced stage of disease and is accompanied by behavioural changes, cognitive impairment, and alterations in sleep–wake cycles that give the disease its name. Although intensified surveillance and control efforts have significantly reduced reported incidence, therapeutic options remain limited and depend on disease stage, with treatment often requiring parenteral administration and careful clinical monitoring [98].

These clinical and pharmacological constraints continue to motivate the identification of parasite-specific vulnerabilities rooted in the distinctive regulatory biology of *T. brucei*.

In this organism, maintenance of parasite identity depends on a constrained regulatory framework in which transcriptional control is largely post-transcriptional and genome organisation assumes a dominant role. This configuration suggests limited functional redundancy, potentially rendering perturbations biologically consequential [99].

Siegel et al. (2008) addressed how epigenetic marks are established in this context by analysing histone H4 acetylation in *T. brucei* [100]. Using mass spectrometry and immunological approaches, they showed that acetylation occurs predominantly at lysine 4 of histone H4 (H4K4), unlike the multiple acetylation sites typically observed in other eukaryotes. Most histone H4 molecules carried this modification, indicating that H4K4ac represents a stable chromatin feature rather than a transient regulatory signal. The non-acetylated form of H4K4 was detected mainly during S phase and was associated with newly synthesised histones, disappearing rapidly after nuclear entry. These results indicate that H4K4 acetylation is established early during chromatin assembly and maintained thereafter, consistent with an epigenetic system tightly coupled to chromatin biogenesis [100].

Building on this concept, Schulz et al. (2015) tested whether recognition of acetylated histones is required to sustain the bloodstream form of *T. brucei* in the mammalian host [101]. Pharmacological inhibition of bromodomain-containing proteins caused marked disruption of gene expression, including repression of bloodstream-form genes and partial activation of transcripts normally associated with the procyclic stage. This response did not correspond to physiological differentiation but rather to a transcriptionally unstable state in which features of distinct life-cycle stages coexisted.

These transcriptional alterations translated into clear functional consequences. Inhibited parasites displayed progressive reduction in the dominant variant surface glycoprotein (VSG), activation of normally silent VSG expression sites, and impaired antibody internalisation at the cell surface, indicating compromised immune evasion [102]. These effects were reversible upon inhibitor removal, with restoration of VSG expression and re-establishment of the originally active expression site, supporting the interpretation of an epigenetic imbalance rather than terminal differentiation.

Direct binding of the inhibitor to the bromodomains Bdf2 and Bdf3, together with phenotypic recapitulation by genetic approaches, linked bromodomain function to the maintenance of the bloodstream form. Structural analyses further showed that the Bdf2 bromodomain differs from human counterparts, and bromodomain inhibition reduced parasite fitness in a murine infection model.

At the genome-wide level, this dependence on chromatin reading is reinforced by higher-order nuclear organisation. Vellmer et al. (2022) demonstrated that the *T. brucei* genome is partitioned into chromatin domains that correlate with transcriptional activity, with active and silent regions occupying distinct nuclear environments in bloodstream forms [103]. Comparisons with procyclic parasites revealed extensive remodelling of chromatin interactions associated with stage-specific transcriptional changes. Actively transcribed VSG expression sites localised to nuclear regions permissive to high transcriptional output, whereas silent VSGs remained spatially segregated. Thus, transcriptional switching was accompanied by coordinated changes in chromatin contacts, directly linking nuclear architecture to transcriptional competence.

Against this backdrop, Poli et al. (2023) systematically addressed a key unresolved issue emerging from bromodomain inhibition studies: the apparent mismatch between the strong differentiation phenotype induced by the bromodomain inhibitor compound (**27**) and its only micromolar affinity for the parasite bromodomains TbBdf2 and TbBdf3 [104]. The central research question was to determine which proteins and which structural features of compound (**27**) are responsible for the transcriptional reprogramming observed in the bloodstream form.

To address this, the authors adopted a rational medicinal chemistry approach, synthesising a series of compound (**27**) derivatives that preserve the core pharmacophore while incorporating functional linkers compatible with chemoproteomic strategies. These compounds were evaluated in phenotypic differentiation assays, including induction of procyclin (EP1), surface coat remodelling, and inhibition of parasite growth, alongside biophysical binding assays (SPR and ITC) against TbBdf2 and TbBdf3.

**Figure 7:**
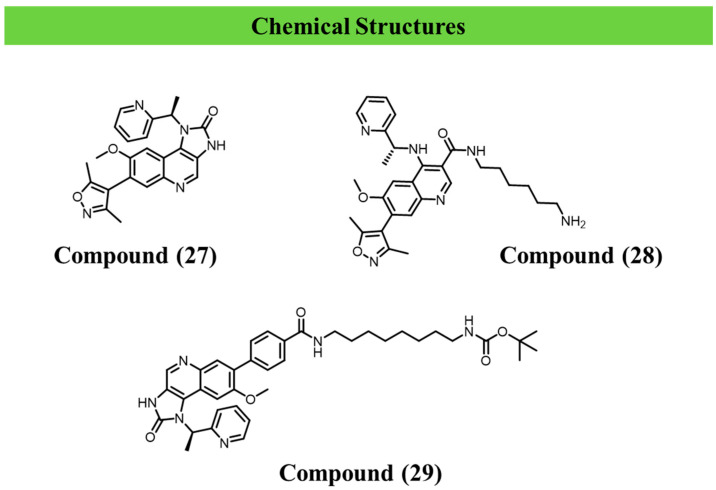
Chemical structures of I-BET151 and rationally designed derivatives used to interrogate bromodomain-dependent differentiation in *T. brucei*. Functionalised analogues preserve the core pharmacophore while enabling chemoproteomic interrogation, revealing strong phenotypic effects despite weak binding to TbBdf2 and TbBdf3.

Strikingly, selected derivatives—most notably compound (**28**) and compound (**29**)—robustly phenocopied the effects of compound (**27**), promoting loss of bloodstream-form identity and reduced parasite fitness, despite exhibiting only weak and transient interactions with the known bromodomain targets. Collectively, these findings underscore the extreme sensitivity of the *T. brucei* epigenetic system to perturbations in chromatin reading, while simultaneously exposing the intrinsic limitations of strategies based solely on reversible inhibition, as the precise molecular determinants underlying the phenotype remain incompletely defined [104,105] (Figure 7).

The compounds described in this section are summarised in Table 5.

These studies establish that epigenetic regulation in *T. brucei* is both central to bloodstream-form identity and highly vulnerable to disruption. Even partial interference with chromatin reading is sufficient to destabilise transcriptional programmes and compromise parasite fitness; however, the effects of classical bromodomain inhibitors remain reversible and constrained by selectivity.

Within this conceptual framework, TPD has been explored as a potential alternative to reversible inhibition in trypanosomatid parasites. The authors argue that *T. brucei’s* pronounced reliance on a limited set of essential chromatin-associated regulators could, in principle, favour degradation-based strategies. However, substantial translational barriers remain, including incomplete characterisation of the parasite ubiquitin–proteasome system, the lack of validated ligands for parasite-specific E3 ligases, and the physicochemical constraints inherent to bifunctional degraders. Thus, while TPD provides a coherent conceptual extension of chromatin vulnerability, its application in *T. brucei* remains exploratory and requires rigorous validation before therapeutic feasibility can be inferred [105].

Taken together, these findings indicate that the strongest current rationale for TPD in *T. brucei* lies in the biological importance of chromatin-associated regulators, rather than in the existence of a validated degradation platform. Although induced protein depletion can generate strong phenotypic effects, this should not be taken as evidence that medicinally useful small-molecule degraders are already available. In particular, the direct extrapolation of IMiD-based recruitment from mammalian systems remains problematic: a degrader that primarily engages host CRBN would raise clear selectivity concerns, while equivalent parasite recruiters have not yet been convincingly demonstrated. Accordingly, future progress will depend not only on defining relevant targets but also on identifying degradation architectures that are compatible with trypanosome biology and distinct enough from host ubiquitin-ligase systems to support selective intervention.

### 5.3. Leishmaniasis

Leishmaniasis is one of the 17 neglected tropical diseases listed by WHO [106]. It is transmitted by phlebotomine sandflies, primarily *Phlebotomus* spp. in the Old World and *Lutzomyia* spp. in the New World, and is caused by protozoan parasites of the genus *Leishmania*. Clinically, the disease is classically divided into two major forms, cutaneous leishmaniasis (CL) and visceral leishmaniasis (VL), the latter representing the most severe and potentially fatal manifestation [107]. Despite the longstanding use of pentavalent antimonials such as meglumine antimoniate, therapeutic options remain limited due to toxicity and variable efficacy, and the global burden of disease persists in the absence of safe and broadly effective treatments [107].

*Leishmania* parasites exhibit a digenetic life cycle involving zoonotic or anthroponotic transmission, depending on the infecting species. During a blood meal, an infected female sandfly inoculates parasites into the mammalian host, where infection may remain localised in the skin or disseminate to internal organs. In macrophages, parasites encounter oxidative stress, including reactive species (ROS), a key effector of innate immunity [108]. Several antiparasitic chemotypes exploit redox imbalance as a therapeutic mechanism [109].

However, drug resistance and the intrinsic plasticity of kinetoplastids remain major obstacles, reinforcing the need to identify new molecular targets beyond redox-dependent pathways, particularly those associated with chromatin regulation. At present, direct evidence for TPD in Leishmania is still lacking; thus, the following studies are discussed primarily to highlight epigenetic target classes that may support future degrader development.

Epigenetic regulation has emerged as a potential vulnerability in kinetoplastids. Comparative analyses of deacetylase repertoires indicate that *Leishmania* encodes class I and class II HDACs as well as sirtuin-like enzymes, and multiple putative HDAC genes have been annotated in *L. braziliensis* relative to human orthologues [110]. Accordingly, several screening efforts have evaluated HDAC inhibitors (HDACi) for antileishmanial activity. For example, hydroxamate-based series originally developed against helminth HDAC targets have been repurposed in macrophage infection models, yielding candidate compounds with measurable activity against *L. braziliensis*. Similarly, aminophenylhydroxamate and aminobenzylhydroxamate derivatives have demonstrated activity in promastigotes, alongside in vitro profiling against human HDAC isoforms, supporting the broader premise that parasite HDAC pathways may be pharmacologically tractable [111].

However, contrasting results have been reported for clinically approved HDAC inhibitors. Chua et al. (2017) tested four FDA-approved HDACi (vorinostat, belinostat, panobinostat, and romidepsin) across several parasites and reported that none of these agents exhibited activity against *Leishmania* promastigotes or amastigotes at the concentrations tested (IC_50_ > 20 µM) [112]. These findings suggest that global histone acetylation induced by broad-spectrum HDAC inhibition may be insufficient to affect parasite viability.

Additionally, aminophenylhydroxamate and aminobenzylhydroxamate derivatives have been evaluated against *L. infantum* and *L. braziliensis*. The authors identified compounds (**30**) and (**31**) as active derivatives. Compound (**30**) exhibited IC_50_ values of 19.6 ± 1.5 µM and 29.3 ± 14.4 µM against *L. infantum* and *L. braziliensis*, respectively. Compound (**31**) exhibited a similar activity profile, with IC_50_ values of 23.5 ± 1.5 µM and 27.2 ± 2.5 µM against *L. infantum* and *L. braziliensis*, respectively [111].

Collectively, these results indicate that modest antileishmanial activity and limited selectivity restrict the translational appeal of hydroxamate HDAC inhibitors [111].

A mechanistic framework that helps reconcile these observations considers the epigenetic system coordinated by writers, erasers, and reader proteins. In contrast to the constrained acetylation architecture described in *T. brucei*, Leishmania displays a highly decorated chromatin landscape, suggesting that vulnerability may reside not in the presence of acetyl marks but in their interpretation by essential reader complexes. In this context, histone acetylation is dynamically regulated and interpreted by bromodomain-containing proteins that recruit transcriptional and chromatin-regulatory machinery.

Consistent with this model, a recent histone post-translational modification (hPTM) atlas in *L. braziliensis* revealed a chromatin landscape enriched in acetylation and methylation marks across canonical histones and variants [113]. Functional studies have identified Bromodomain Factor 5 (BDF5) as essential for parasite viability, with enrichment at transcription start regions and a marked reduction in global transcription following deletion. BDF5 bromodomains bind acetylated histone peptides and can be chemically engaged by small molecules, supporting their druggability [114].

Russell et al. (2023) identified compound (**32**), also known as SGC-CPB30, as a human bromodomain inhibitor with activity against *L. mexicana* and *L. donovani*, exhibiting IC_50_ values of 7.1 and 6.1 µM, respectively [114].

**Figure 8:**
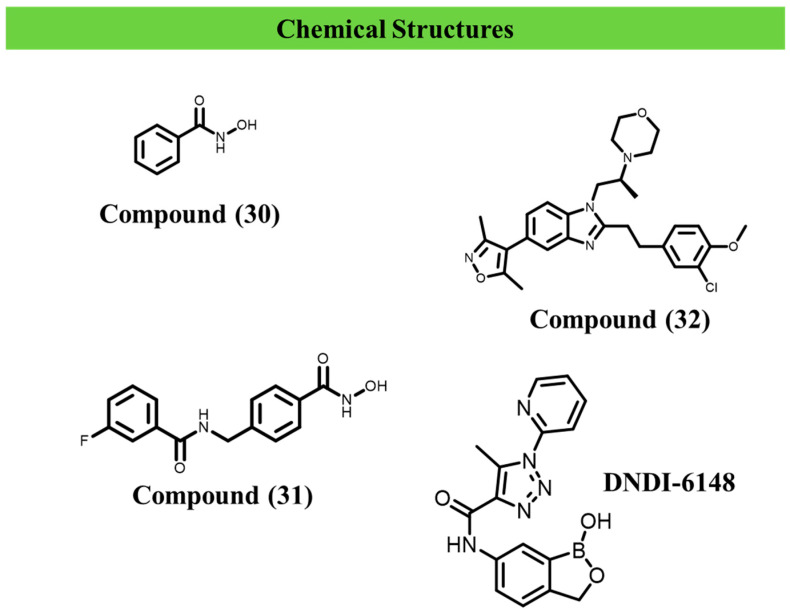
Chemical structures of representative compounds discussed in the Leishmania section, including HDAC inhibitors (**30**) and (**31**), the bromodomain inhibitor compound (**32**), and the CPSF3-targeting benzoxaborole DNDI-6148.

Figure 8 summarises the structures described in this section.

Together, these findings suggest that the limited efficacy of clinically approved HDACi in *Leishmania* may reflect not only differences in target engagement, isoform selectivity, and intracellular access in the parasite, but also the possibility that the most vulnerable node of the parasite epigenetic circuitry resides at the level of acetylation “reading” and recruitment. Consequently, targeting essential bromodomain readers may represent a more mechanistically coherent strategy than targeting eraser enzymes with inhibitors originally optimised for human HDACs.

This limited efficacy contrasts with the activity of HDAC inhibitors in other trypanosomatids, such as *T. brucei* and *T. cruzi*, and likely reflects fundamental differences in gene regulation. Unlike these organisms, where chromatin structure and histone modifications play a central role in controlling transcriptional programmes, Leishmania relies predominantly on post-transcriptional mechanisms, including mRNA stability and translational control. As a result, modulation of histone acetylation may have a reduced impact on gene expression and parasite viability.

This distinction suggests that, in Leishmania, epigenetic modifications are less determinant of transcriptional output and more dependent on downstream regulatory layers, which may explain the lower sensitivity to HDAC inhibition. Instead, targeting epigenetic reader proteins or RNA-binding regulatory complexes may represent a more effective strategy. In this context, CPSF3 (cleavage and polyadenylation specificity factor 3), targeted by benzoxaborole compounds such as DNDI-6148, exemplifies an alternative approach based on disruption of mRNA processing.

Although *Leishmania* possesses ubiquitination machinery and functionally relevant E3 ligases, including HECT-type ligases involved in differentiation and Cullin-1-RING complexes in *L. infantum*, these systems remain insufficiently characterised from a chemical biology perspective, and ligandable recruiters analogous to CRBN or VHL have not yet been established.

Thus, in *Leishmania*, the current rationale for TPD remains primarily target-driven rather than platform-driven. Epigenetic readers and RNA-processing factors provide biologically meaningful vulnerabilities, but the absence of validated parasite-selective recruiters and the intracellular localisation of the parasite still limit immediate translation into degrader-based therapeutics.

The compounds described in this section are summarised in Table 6.

### 5.4. Malaria (Plasmodium falciparum)

Malaria remains one of the most serious infectious diseases worldwide, posing a substantial threat to global public health. It is caused by protozoan parasites of the genus Plasmodium and is transmitted primarily through the bite of infected female Anopheles mosquitoes, although transmission may also occur through blood transfusion or the use of contaminated needles [115].

Among the species infecting humans, *Plasmodium falciparum* and *P. vivax* account for most cases. *P. falciparum*, the most virulent species, predominates in sub-Saharan Africa and is responsible for the highest mortality, whereas *P. vivax* is more widely distributed outside Africa. Early clinical manifestations are often nonspecific and may resemble other febrile illnesses, which can delay diagnosis. If untreated, *P. falciparum* infection may rapidly progress to severe disease and death within 24 h [115].

According to the most recent World Malaria Report, approximately 282 million malaria cases were recorded in 2024, representing an increase of about 9 million cases (3%) compared with 2023, while malaria-related deaths reached approximately 610,000, up from 598,000 in the previous year [115].

At present, direct evidence for TPD in malaria models is still lacking; therefore, the following studies are discussed primarily to identify epigenetic target classes that may support future degrader-based approaches. The continued emergence of resistance to frontline antimalarial therapies highlights the urgent need for new drugs with novel mechanisms of action. In this context, epigenetic regulation has emerged as a promising therapeutic target. HDACs, for example, play essential roles in parasite development and survival. In *P. falciparum*, three main HDAC classes have been identified: (i) PfHDAC1 (PFI1260c), a predominant nuclear class I enzyme; (ii) PfHDAC2 (PF14_0690) and PfHDAC3 (PF10_0078), classified as class II HDACs; and (iii) the class III sirtuins PfSir2A (PF13_0152) and PfSir2B (PF14_0489) [116,117,118].

Although PfSir2A and PfSir2B are involved in the regulation of *var* gene expression and antigenic variation, they are considered nonessential for parasite survival, which reduces their attractiveness as primary drug targets. In contrast, PfHDAC1 is highly conserved among *Plasmodium* species and is implicated in critical biological processes, including gametocytogenesis, schizogony, and hepatocyte invasion. Notably, PfHDAC1 shares approximately 61% sequence similarity with human HDACs, supporting its biological relevance while also emphasising the need for selective inhibition to minimise host toxicity [119].

**Figure 9:**
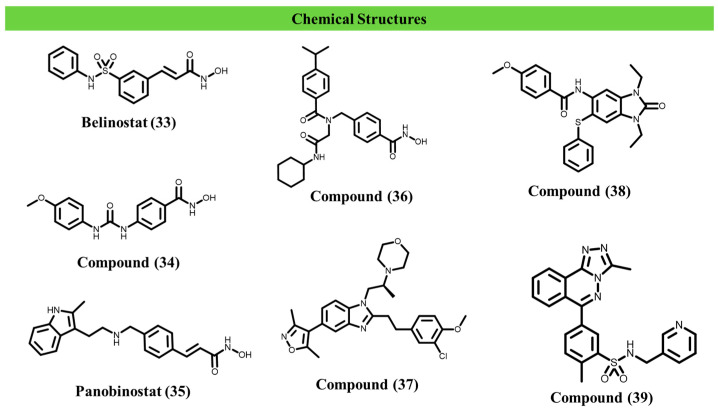
Chemical structures of the compounds (**33**)–(**39**) evaluated against *Plasmodium* spp.

Several medicinal chemistry efforts have explored HDACi as potential antimalarial agents. A series of 1,3-diphenylureido hydroxamate derivatives has also been described as active against *P*. *falciparum*. In their design, a cinnamyl hydroxamate moiety was employed as the zinc-binding group (ZBG) and linker, connected to various *p-*substituted phenylurea groups. To assess the structural determinants of activity, the authors investigated the influence of linker saturation and evaluated analogues lacking the double bond present in the original scaffold. Among the synthesised compounds, derivative (**34**) (Figure 9) exhibited the most favourable antiparasitic profile, combining potent activity with low cytotoxicity, with an IC_50_ of 0.74 µM against P*f*3D7 and an SI > 271 [120].

From a structural perspective, phenyl hydroxamate inhibitors selective for human HDAC6 typically exhibit monodentate coordination to the catalytic Zn^2+^ ion due to steric constraints, whereas more flexible saturated acyl chains tend to maintain bidentate coordination. Importantly, the incorporation of a diphenylurea cap-linker moiety correlated with enhanced antiplasmodial potency compared with cinnamyl or dihydrocinnamyl linkers. Furthermore, replacing cinnamyl hydroxamates improved selectivity, as evidenced by minimal cytotoxicity in HepG2 cells (IC_50_ > 200 µM), contrasting with the higher toxicity observed in earlier cinnamyl-based analogues [120].

In parallel, drug repurposing strategies have provided an alternative pathway for accelerating antimalarial drug discovery. In this context, clinically approved HDAC inhibitors have been evaluated for activity against *Plasmodium knowlesi* [121]. In contrast to findings in other protozoan parasites such as *Leishmania*, several HDAC inhibitors displayed high potency and selectivity against *P. knowlesi*. Belinostat, panobinostat, and vorinostat exhibited IC_50_ values in the low nanomolar range (9–370 nM) and demonstrated 8- to 45-fold selectivity for the parasite over human neonatal foreskin fibroblast (NFF) and HEK293 cells. Romidepsin, although potent, lacked selectivity.

All active compounds induced hyperacetylation of parasite histone H4, confirming on-target epigenetic activity. These findings highlight the potential of drug repurposing to identify antimalarial candidates from scaffolds with established pharmacokinetic and toxicological profiles [121].

Further support for HDAC inhibition as a viable antimalarial approach comes from studies on clinical *P. falciparum* isolates. Koehne and colleagues evaluated 85 clinical isolates from patients with uncomplicated malaria in Gabon, assessing their susceptibility to a panel of HDAC inhibitors and to established antimalarial agents. Among the tested compounds, peptide-based HDAC inhibitor (**36**) exhibited remarkable potency, with a mean IC_50_ of 4 nM and high selectivity for the parasite [119].

Beyond HDACs, other epigenetic regulators, such as bromodomain-containing proteins, have also been investigated as antimalarial targets. Evaluation of bromodomain inhibitors against asexual blood-stage *P. falciparum* identified three active compounds: compound (**37**) (IC_50_ = 10.03 ± 0.32 µM), compound (**38**) (IC_50_ = 11.28 ± 2.00 µM), and compound (**39**) (IC_50_ = 11.80 ± 3.06 µM) (Figure 9). These findings further support epigenetic modulation as a rational and innovative therapeutic strategy [121].

The compounds described in this section are summarised in Table 7.

Taken together, these findings indicate that the strongest current rationale for TPD in malaria lies not in the existence of a validated degrader platform, but in the biological importance of chromatin-associated regulators and the clear vulnerability of the parasite to epigenetic perturbation. At present, however, direct evidence for small-molecule degraders in *Plasmodium* remains limited, and most available data still derive from catalytic inhibition rather than regulated protein depletion. This distinction is important, because the main translational bottleneck is not simply target identification, but the definition of a degradation architecture compatible with parasite biology.

In particular, the lack of chemically validated parasite recruiters and the uncertain transferability of host ubiquitin-ligase systems argue against treating canonical PROTAC design as an immediately established route for antimalarial drug discovery. Thus, while TPD represents a conceptually attractive extension of current epigenetic strategies, future progress will depend on demonstrating that selective protein degradation can be achieved in malaria models with mechanisms that are both biologically relevant and pharmacologically credible.

## 6. Perspectives and Future Directions

Although TPD has advanced substantially in oncology, its translation to infectious diseases remains limited. This disparity reflects not only differences in translational maturity and investment but also biological differences between cancer and infection. Host–pathogen interactions are inherently dynamic, involving stage-specific gene regulation, transcriptional reprogramming, and adaptive responses that complicate target selection and pharmacological design.

Proof-of-concept studies, such as BacPROTAC-mediated engagement of bacterial Clp proteases, demonstrate the feasibility of hijacking endogenous degradation systems. However, most efforts have focused on model substrates or essential enzymes, with comparatively little attention given to epigenetic regulators that coordinate transcriptional adaptation.

In protozoan parasites, incomplete characterisation of ubiquitin-proteasome components and alternative degradation pathways further constrains rational degrader development. In addition, functional redundancy among chromatin-associated proteins may buffer the impact of degrading a single factor, suggesting that combinatorial strategies could be required.

Host-directed approaches also demand careful evaluation, as sustained depletion of regulatory proteins may alter immune homeostasis.

Host defence, additional hurdles include the identification of selective pathogen-directed ligands, optimisation of physicochemical properties compatible with intracellular delivery, and the structural conservation of many epigenetic modules between pathogen and host.

Yet these same biological features reveal a significant opportunity. In many infectious contexts, pathogenic phenotypes depend not solely on catalytic activity but on the abundance, stability, or assembly of regulatory complexes. Direct modulation of protein levels may therefore disrupt infection-associated programmes more profoundly than reversible inhibition alone.

Organism-specific regulatory architectures further illustrate this rationale. In *Trypanosoma brucei*, where antigenic variation and stage differentiation rely on tightly regulated chromatin organisation and multiprotein transcriptional complexes, degradation of non-enzymatic chromatin scaffolds or bromodomain-containing readers may disrupt coordinated gene expression programmes beyond the scope of single-enzyme inhibition.

In *Leishmania* spp., where transcriptional control is largely post-transcriptional and chromatin organisation contributes to developmental plasticity, targeting regulatory adaptors rather than individual enzymatic activities may offer a strategy to perturb differentiation states associated with persistence.

In Mtb, chromatin-associated proteins such as Lsr2 and related nucleoid-structuring factors regulate stress adaptation and latency; selective degradation of such regulatory hubs could, in principle, interfere with persistence phenotypes that are only partially sensitive to conventional enzymatic inhibition. These examples are summarised in Table 8, which highlights candidate targets, potential degradation machineries, and key translational bottlenecks across infectious systems.

In this context, a shift from occupancy-driven pharmacology to abundance-driven modulation may broaden anti-infective drug discovery paradigms. Moreover, degradation technologies offer a valuable platform for target validation, enabling assessment of protein essentiality beyond catalytic blockade and providing mechanistic resolution in systems where regulatory plasticity underlies persistence.

Progress in this area will depend on integrating chemical biology, structural insight, and infection-specific target prioritisation, while also overcoming major translational barriers. Efficient intracellular delivery remains a limitation, particularly in infections in which degraders must access infected cells and, in some cases, specific subcellular compartments. Achieving selectivity in host–pathogen systems is also critical, as many proposed targets are structurally conserved or functionally shared, increasing the risk of off-target effects and perturbing essential host processes.

More broadly, the translation of TPD to infectious diseases should be viewed through an integrated framework that links four interdependent requirements: target accessibility, compatibility with degradation machinery, degrader developability, and infection-context validation. A candidate target must be reachable within the relevant host, pathogen, or host–pathogen compartment; the recruited proteolytic pathway must be present, active, and selectively engageable in that biological setting; the degrader must display physicochemical properties compatible with permeability, metabolic stability, tissue exposure, and intracellular delivery; and the resulting degradation event must produce a durable anti-infective phenotype in infection-relevant models. Failure at any one of these levels may disconnect target ligandability from therapeutic feasibility.

Therefore, future anti-infective TPD programmes should prioritise not only biologically essential targets, but also the cellular route by which degraders reach them and the disease context in which protein depletion is expected to translate into pharmacological benefit.

Additional barriers include the physicochemical complexity of degrader molecules, the need for validation in physiologically relevant in vivo models, and the adaptive nature of infectious systems, which may require multi-target or combinatorial strategies. By embracing context-dependent modulation of protein abundance, degradation-based strategies may help establish a new conceptual framework for anti-infective drug discovery, grounded in the dynamic regulation of pathogenic proteomes and aligned with the biological plasticity that sustains infection.

## 7. Conclusions

Epigenetic regulation has emerged as a central determinant of infection biology, influencing pathogen adaptability, immune modulation, and therapeutic response. In bacteria, mycobacteria, viruses, and kinetoplastid parasites, chromatin-associated mechanisms contribute to persistence, virulence, and resistance phenotypes that are not fully addressed by classical enzymatic inhibition. Strategies based solely on occupancy often fail to disrupt protein complexes or dosage-sensitive regulators that control transcriptional programmes during infection.

TPD offers a distinct approach by directly modulating protein abundance. By engaging proteasomal, lysosomal, or autophagy-dependent pathways, degradation platforms may expand the range of tractable targets to include epigenetic readers, writers, erasers, and pathogen-derived effectors. Early examples in bacterial systems and virus-associated host factors demonstrate feasibility, although applications in infectious and neglected diseases remain limited.

Key obstacles include the identification of selective ligands, constraints on intracellular delivery, incomplete understanding of pathogen- or parasite-compatible degradation machinery, and limited translational investment in neglected conditions. Despite these challenges, combining epigenetic insight with protein-level intervention provides a rational strategy to interfere with persistence and adaptive resistance.

Advancing degradation-based approaches beyond oncology will require pathogen-informed design, context-specific target validation, and integration of epigenetic insight with protein-level modulation. In infectious contexts characterised by regulatory plasticity and adaptive persistence, reshaping pathogenic proteomes may prove as important as inhibiting catalytic functions, thereby expanding the conceptual and therapeutic boundaries of anti-infective drug discovery.

## Figures and Tables

**Table 1:** Advantages and limitations of TPD strategies.

Advantages	
**Event-driven pharmacology**	Transient target engagement is sufficient to induce degradation.
**Sustained biological effect**	Functional effects persist after dissociation of the degrader.
**Elimination of non-catalytic functions**	Degradation removes the target protein irrespective of its functional roles.
**Catalytic mechanism**	A single molecule can induce degradation of multiple copies of the target protein.
**Limitations**	
**Dependence on ternary complex formation**	Efficient degradation requires productive formation and sufficient stability of a ternary complex.
**Hook effect**	High concentrations reduce degradation efficiency due to binary complex formation.
**Physicochemical and pharmacokinetic limitations**	High molecular weight, increased polarity, low permeability, and limited stability may compromise bioavailability, tissue exposure, and intracellular delivery.
**Dependence on compatible degradation machineries**	Degradation depends on the availability, accessibility, and compatibility of ligases or alternative proteolytic systems.
**Target accessibility/subcellular compartmentalisation**	Efficient degradation depends on the ability of the degrader to access targets located in specific cellular or host–pathogen compartments.
**Limited knowledge of exploitable degradation machineries in pathogens**	In many pathogens, suitable ligases or alternative proteolytic systems remain poorly characterised, limiting rational degrader design.
**Tissue exposure/intracellular delivery**	Degrader activity may be compromised by insufficient penetration into infected tissues and limited access to intracellular microorganisms or pathogen-containing compartments.
**Scarcity of validated targets**	Many infection-relevant targets remain biologically interesting but insufficiently validated for degradation-based therapeutic intervention, particularly in neglected tropical diseases.

**Table 2:** Summary of compounds evaluated in tuberculosis and mycobacterial models.

Compound	Biological Activity	Mechanism	Biological Effect	Strategy	Reference
(**1**)	MIC > 250 µM No direct anti-Mtb activity	Host–cell activation	Reduced intracellular Mtb burden by increasing mediators (LL-37, HBD-2, SOD3, iNOS)	Potential candidates for AUTAC and ATTEC-based strategies	[57]
(**2**)					
(**3**)					
(**4**)					
(**5**)	K_d_ = 10 µM	ClpC-mediated degradation of BRDTBD1	Approximately 50% reduction in target protein (after 30 min)	BacPROTAC	[64]
(**6**)	K_d_ = 20 µM		Approximately 25% reduction in target protein (after 2 h)		

K_d_ = dissociation constant.

**Table 3:** Overview of epigenetic modulators and degraders in viral infections.

Compound	Biological Activity	Target	Mechanism	Biological Effect	Strategy	Reference
(**7**)	250 nM	HDAC	Enzymatic inhibition	Modulates histone acetylation	Classic inhibitor	[75]
(**8**)	40 nM	HDAC-3	HDAC-3 degradation	Impairs latency establishment	PROTAC	
(**9**)	0.1–0.5 µM	HDAC-6	HDAC6 degradation	Reduced viral replication		[81]

**Table 4:** Summary of compounds targeting epigenetic regulation in *T. cruzi*. Compounds (**10**)–(**15**) correspond to enzymatic modulators of acetylation-related pathways, including HDAC and HAT inhibitors, whereas compounds (**16**)–(**26**) are bromodomain ligands targeting TcBDF2 or TcBDF3. Activity values are reported as described in the original studies and may refer to biochemical binding, antiparasitic activity, or host–cell toxicity, depending on the experimental context.

Compound	Activity	Target	Biological Effect	Reference
(**10**)	EC_50_ = 4 µM	HDAC	High antiparasitic potency, but low selectivity	[90]
(**11**)	EC_50_ = 7 µM; SI = 5.7	HDAC6/8	Improved selectivity with retained antiparasitic activity	
(**12**)	EC_50_ = 13.9 µM	HDAC	Moderate antiparasitic activity with low cytotoxicity	
(**13**)	IC_50_ = 7.0 µM	HAT	Reduced epimastigote proliferation and intracellular amastigote replication	[91]
(**14**)	IC_50_ = 14 µM		Parasite growth inhibition associated with high host–cell toxicity	
(**15**)	>100 µM		Limited antiparasitic effect despite mitochondrial alterations	
(**16**)	K_d_ = 1.91 µM	TcBDF2	Biochemical binding without significant cellular activity	[92]
(**17**)	K_d_ = 1.33 µM			
(**18**)	K_d_ = 2.41 µM			
(**19**)	K_d_ = 1.58 µM			
(**20**)	K_d_ = 3.52 µM			
(**21**)	K_d_ = 3.31 µM			
(**22**)	K_d_ = 2.81 µM			
(**23**)	K_d_ = 1.7 µM; IC_50_ = 2.4 µM	TcBDF3	Active across parasite stages with low host–cell cytotoxicity	[93,94]
(**24**)	K_d_ = 4.0 µM; IC_50_ = 8.4 µM		Improved cellular potency, especially against trypomastigotes	
(**25**)	K_d_ = 136 µM		Weak binding; not prioritised	
(**26**)	K_d_ = 4.8 µM; CC_50_ = 315.6 µM		Low host–cell toxicity with lower overall antiparasitic potency	

**Table 5:** Summary of compounds targeting *T. brucei* epigenetic regulation.

Compound	Activity	Target	Mechanism	Biological Effect	Reference
(**27**)	Qualitative phenotypic activity	Bdf2/Bdf3	Bromodomain inhibition	Transcriptional reprogramming	[104]
(**28**)					
(**29**)					

**Table 6:** Summary of compounds with antileishmanial activity.

Compound	Biological Activity	Target	Mechanism	Reference
(**30**)	IC_50_ = 19.6 ± 1.5 µM ^†^; 29.3 ± 14.4 µM ^δ^	HDAC	Enzymatic inhibition	[111]
(**31**)	IC_50_ = 23.5 ± 1.5 µM ^†^; 27.2 ± 2.5 µM ^δ^			
(**32**)	IC_50_ = 7.1 µM ^§^; 6.1 µM ^#^	Bromodomain reader (BDF5-related)	Bromodomain inhibition	[114]

^†^  *L. infantum*; ^δ^ *L. braziliensis*; ^§^ *L. mexicana*; ^#^ *L. donovani*.

**Table 7:** Summary of compounds with antimalarial activity.

Compound	Activity	Target	Mechanism	Reference
(**33**)	IC_50_ = 9–370 nM	HDAC (*P. knowlesi*)	Enzymatic inhibition	[120,121]
(**34**)	IC_50_ = 0.74 µM			
(**35**)	IC_50_ = 9–370 nM			
(**36**)	IC_50_ = 4.0–14.7 nM (anti-plasmodial activity)	HDAC	Phenotypic assays against *P. falciparum* strains	[119]
(**37**)	IC_50_ = 10.03 ± 0.32 µM	Bromodomain	Bromodomain inhibition	[121]
(**38**)	IC_50_ = 11.28 ± 2.00 µM			
(**39**)	IC_50_ = 11.80 ± 3.06 µM			

**Table 8:** Candidate targets, potential degradation machineries, current evidence, and translational bottlenecks for TPD strategies in infectious diseases.

Disease/ Pathogen	Candidate Targets	Potential Degradation Machinery/Recruiter *	Current Status	Main Bottleneck	References
AMR bacteria	Virulence factors; bacterial methyltransferases	LYTAC-like concepts; bacterial proteases	Mostly conceptual	Functional validation in bacterial systems	[30,31,32,33,34,35,36,37,38,39,40,41,42,43,44,45]
Tuberculosis/*Mtb*	Lsr2; nucleoid-associated proteins; Clp substrates	ClpC1–ClpP1P2	BacPROTAC proof-of-concept evidence	Macrophage delivery; native target validation	[57,58,59,60,61,62,63,64]
HIV-1	HDAC1/2/3; BRD4	Host E3 ligases; CRBN; Vpr/DCAF1-inspired recruiters	Cellular proof-of-concept evidence	Latency complexity; selectivity	[77,78,79,80,81]
IAV	HDAC6	CRBN-based PROTAC	Cellular proof-of-concept evidence	Full infection dynamics unclear	[82,83]
*T. cruzi*	TcBDF3; TcHsp90; TcTopoIIα; cruzain	Parasite UPS; non-canonical TPD possible	Strong target rationale; limited degrader evidence	No validated parasite recruiters	[86,87,88,89,90,91,92,93,94,95,96]
*T. brucei*	Bdf2/Bdf3; chromatin readers	Putative parasite UPS; genetic depletion tools	Strong epigenetic vulnerability	Degradation architecture unresolved	[97,98,99,100,101,102,103,104,105]
*Leishmania* spp.	BDF5; CPSF3; RNA-processing regulators	HECT-type E3s; CRL1; non-canonical TPD possible	Epigenetic target evidence; no direct TPD	Ligandable E3 recruiters absent	[106,107,108,109,110,111,112,113,114]
Malaria/*Plasmodium* spp.	PfHDAC1; bromodomain proteins	Unknown parasite-compatible recruiters	Strong inhibitor data; no validated degraders	Recruiter validation; delivery	[115,116,117,118,119,120,121]

* Potential degradation machinery/recruiter refers to biologically plausible or experimentally discussed degradation systems and does not necessarily imply chemically validated ligandable recruiters.

## Data Availability

No new data were created or analyzed in this study. Data sharing is not applicable to this article.

## Abbreviations

The following abbreviations are used in this manuscript:

AMR	Antimicrobial-Resistant Bacteria
ATP	Adenosine triphosphate
ATPase	Adenosine triphosphatase
ATTEC	Autophagosome-Tethering Compound
AUTACs	Autophagy Targeting Chimera
BacPROTAC	Bacterial Proteolysis Targeting Chimera
BRDTBD1	Bromodomain and TBC Domain Containing 1
BDF5	Bromodomain Factor 5
CC_50_	Concentration required to reduce cell viability by 50%
CL	Cutaneous leishmaniasis
Clp	Caseinolytic Proteases
DNA	Deoxyribonucleic acid
DNMT-DNA	methyltransferase family
EC_50_	Concentration required to achieve 50% of the maximal effect
EIS	Enhanced intracellular survival
ESBL	Extended-spectrum *β*-lactamase
XDR-TB	Extensively Drug-Resistant
FDA	Food and Drug Administration
H_37_Rv	Reference laboratory strain of *Mycobacterium tuberculosis*
H4K4ac	Histone H4 lysine 4 acetylation
HDAC	Histone deacetylase
HDACi	Histone deacetylase inhibitors
HAT	histone acetyltransferases
HIV	Human immunodeficiency virus
Hla	Alpha-hemolysin
HU	Histone-like protein HU
IAV	Influenza A virus
LRA	latency-reversing agents
LYTAC	Lysosome Targeting Chimera
MDR-TB	Multidrug-Resistant
Mtb	*Mycobacterium tuberculosis*
NFF	Neonatal Foreskin Fibroblast
NTD	Neglected Tropical Diseases
PKF	Secreted serine protease of *Acinetobacter baumannii*
PROTAC	Protein Target Chimera
PVL	Panton-Valentine leukocidin
RNA	Ribonucleic acid
ROS	Reactive oxygen species
SI	Selective Index
TPD	Targeted Protein Degradation
TB	Tuberculosis
VSG	variant surface glycoprotein
vRNP	viral ribonucleoprotein
VL	Visceral Leishmaniasis
WHO	World Health Organization
ZBG	zinc-binding group
